# Supplementary material for: Health Literacy and Self-Care in Patients with Chronic Illness: A Systematic Review and Meta-Analysis Protocol
Source: Healthcare (Basel). 2024 Mar 31;12(7):762. doi: 10.3390/healthcare12070762 (PMC11011384; doi:10.3390/healthcare12070762)
Supplement: Supplementary file 1 [file healthcare-12-00762-s001.zip › healthcare-2888560-supplementary.pdf]

Table S1: PubMed search string sample

Search string
("Chronic Disease"[MeSH Terms] OR "chronic disease*"[Title/Abstract] OR "chronic illness*"[Title/Abstract] OR "chronic condition*"[Title/Abstract] OR "Chronically Ill"[Title/Abstract] OR "noncommunicable diseases"[MeSH Terms] OR "noncommunicable disease*"[Title/Abstract] OR "non communicable disease*"[Title/Abstract] OR "non-communicable disease*"[Title/Abstract] OR "non infectious disease*"[Title/Abstract] OR "non-infectious disease*"[Title/Abstract] OR "noninfectious disease*"[Title/Abstract] OR "non communicable chronic disease*"[Title/Abstract] OR "non-communicable chronic disease*"[Title/Abstract] OR "CD"[Title/Abstract] OR "NCD"[Title/Abstract] OR "Hypertension"[MeSH Terms] OR "hypertensi*"[Title/Abstract] OR "high blood pressure*"[Title/Abstract] OR "essential hypertension*"[Title/Abstract] OR "primary hypertension*"[Title/Abstract] OR "Isolated Systolic Hypertension"[Title/Abstract] OR "Isolated Systolic"[Title/Abstract] OR "hypertension*"[Title/Abstract] OR "systolic hypertensi*"[Title/Abstract] OR "Coronary Disease"[Title/Abstract] OR "coronary disease*"[Title/Abstract] OR "coronary heart disease*"[Title/Abstract] OR "coronary artery disease*"[Title/Abstract] OR "left main coronary artery disease*"[Title/Abstract] OR "left main disease*"[Title/Abstract] OR "left main coronary disease*"[Title/Abstract] OR "coronary arterioscleros*"[Title/Abstract] OR "CAD"[Title/Abstract] OR "arterioscleros*"[Title/Abstract] OR "Heart failure"[Title/Abstract] OR "Cardiac Failure"[Title/Abstract] OR "Heart Decompensation"[Title/Abstract] OR "Right-Sided Heart"[Title/Abstract] OR "Right Sided Heart Failure"[Title/Abstract] OR "Myocardial Failure"[Title/Abstract] OR "Congestive Heart Failure"[Title/Abstract] OR "left sided heart failure"[Title/Abstract] OR "left-sided heart failure"[Title/Abstract] OR "CHF"[Title/Abstract] OR "HF"[Title/Abstract] OR "arthriti*"[Title/Abstract] OR "polyarthriti*"[Title/Abstract] OR "oligoarthriti*"[Title/Abstract] OR "osteoarthriti*"[Title/Abstract] OR "renal insufficiency, chronic"[MeSH Terms] OR "chronic renal insufficienc*"[Title/Abstract] OR "chronic kidney insufficienc*"[Title/Abstract] OR "chronic kidney disease*"[Title/Abstract] OR "chronic renal disease*"[Title/Abstract] OR "CKD"[Title/Abstract] OR "Chronic Kidney Failure"[Title/Abstract] OR "end stage kidney disease"[Title/Abstract] OR "end-stage kidney disease"[Title/Abstract] OR "end stage renal disease"[Title/Abstract] OR "end-stage renal disease"[Title/Abstract] OR "end stage renal failure"[Title/Abstract] OR "end-stage renal failure"[Title/Abstract] OR "Chronic Renal Failure"[Title/Abstract] OR "ESRD"[Title/Abstract] OR "stroke*"[Title/Abstract] OR "cerebrovascular accident*"[Title/Abstract] OR "CVA"[Title/Abstract] OR "CVAs"[Title/Abstract] OR "Cerebrovascular Apoplexy"[Title/Abstract] OR "brain vascular accident*"[Title/Abstract] OR "cerebrovascular stroke*"[Title/Abstract] OR "Apoplexy"[Title/Abstract] OR "cerebral stroke*"[Title/Abstract] OR "acute stroke*"[Title/Abstract] OR "acute cerebrovascular accident*"[Title/Abstract] OR "asthma*"[Title/Abstract] OR "Bronchial Asthma"[Title/Abstract] OR "chronic obstructive pulmonary disease*"[Title/Abstract] OR "chronic obstructive lung disease*"[Title/Abstract] OR "COAD"[Title/Abstract] OR "COPD"[Title/Abstract] OR "chronic obstructive airway disease*"[Title/Abstract] OR "chronic obstructive pulmonary disease"[Title/Abstract] OR "chronic airflow obstruction*"[Title/Abstract] OR "Diabetes Mellitus Type 2"[Title/Abstract] OR "noninsulin-dependent diabetes mellitus"[Title/Abstract] OR "noninsulin dependent diabetes mellitus"[Title/Abstract] OR "ketosis-resistant diabetes mellitus"[Title/Abstract] OR "ketosis resistant diabetes mellitus"[Title/Abstract] OR "non-insulin dependent diabetes mellitus"[Title/Abstract] OR "noninsulin dependent diabetes mellitus"[Title/Abstract] OR "Stable Diabetes Mellitus"[Title/Abstract] OR "Type II Diabetes Mellitus"[Title/Abstract] OR "NIDDM"[Title/Abstract] OR "maturity-onset diabetes mellitus"[Title/Abstract] OR "maturity onset diabetes mellitus"[Title/Abstract] OR "MODY"[Title/Abstract] OR "Type 2 Diabetes Mellitus"[Title/Abstract] OR "maturity onset diabetes"[Title/Abstract] OR "maturity-onset diabetes"[Title/Abstract] OR "Type 2 Diabetes"[Title/Abstract] OR "adult onset diabetes mellitus"[Title/Abstract] OR "adult onset-diabetes mellitus"[Title/Abstract] OR "T2DM"[Title/Abstract] OR "diabetes mellitus type II"[Title/Abstract] OR "Essential Hypertension"[MeSH Terms] OR "Coronary Artery Disease"[MeSH Terms] OR "Arteriosclerosis"[MeSH Terms] OR "Arthritis"[MeSH Terms] OR "Stroke"[MeSH Terms] OR "Asthma"[MeSH Terms] OR "pulmonary disease, chronic obstructive"[MeSH Terms] OR "Diabetes Mellitus"[MeSH Terms] OR "diabetes mellitus, type 2"[MeSH Terms] OR "Insulin Resistance"[MeSH Terms]) AND ("Health Literacy"[MeSH Terms] OR "Health Literacy"[Title/Abstract] OR "literacies"[Title/Abstract] OR "information literac*"[Title/Abstract] OR "print literacy"[Title/Abstract] OR "communication literacy"[Title/Abstract] OR "information seeking"[Title/Abstract] OR "information-seeking skills"[Title/Abstract] OR "HL"[Title/Abstract] OR "educational status"[Title/Abstract] OR "numeracy"[Title/Abstract] OR "health knowledge"[Title/Abstract] OR "health attitude"[Title/Abstract] OR "health understanding"[Title/Abstract] OR "health practice"[Title/Abstract] OR "health information management"[MeSH Terms] OR "health information management"[Title/Abstract] OR "health communication"[MeSH Terms] OR "health communication"[Title/Abstract] OR "digital health literacy"[Title/Abstract] OR "eHL"[Title/Abstract] OR "e-HL"[Title/Abstract] OR "digital health competence"[Title/Abstract] OR "e-health literacy"[Title/Abstract] OR "health technology literacy"[Title/Abstract] OR "digital proficiency"[Title/Abstract] OR "Information Literacy"[MeSH Terms]) AND ("Self-management"[MeSH Terms] OR "self manag*"[Title/Abstract] OR "self manag*"[Title/Abstract] OR "self-care"[MeSH Terms] OR "self-car*"[Title/Abstract] OR "self car*"[Title/Abstract] OR "self monitor*"[Title/Abstract] OR "self-administration"[Title/Abstract] OR "self-medication"[Title/Abstract] OR "self medication"[Title/Abstract] OR "compliance"[Title/Abstract] OR "patient compliance"[Title/Abstract] OR "Patient Adherence"[Title/Abstract] OR "Patient Cooperation"[Title/Abstract] OR "client compliance*"[Title/Abstract] OR "Client Adherence"[Title/Abstract] OR "treatment compliance*"[Title/Abstract] OR "therapeutic compliance*"[Title/Abstract] OR "patient non compliance"[Title/Abstract] OR "patient non-compliance"[Title/Abstract] OR "Patient Noncompliance"[Title/Abstract] OR "Patient Nonadherence"[Title/Abstract] OR "patient non adherence"[Title/Abstract] OR "patient non-adherence"[Title/Abstract] OR "non adherent patient*"[Title/Abstract] OR "non adherent patient*"[Title/Abstract] OR "treatment adherence"[Title/Abstract] OR "empowerment*"[Title/Abstract] OR "self-care maintenance"[Title/Abstract] OR "self-care monitoring"[Title/Abstract] OR "self-care management"[Title/Abstract] OR "self-care maintenance"[Title/Abstract] OR "self-care monitoring"[Title/Abstract] OR "self-care management"[Title/Abstract] OR "self care behavi*"[Title/Abstract] OR "self-care behavi*"[Title/Abstract] OR "self-efficacy"[MeSH Terms] OR "self-efficacy"[Title/Abstract] OR "self efficacy"[Title/Abstract] OR "empowerment"[MeSH Terms])

Table S2: Sample table of study characteristics

Author(s)	Year of publication	Aim(s)	Study location	Sample characteristics	Type of chronic illness	Study design	Study duration	HL measure(s)	Self-care measure(s)	Main study results	Risk of bias
…	…	…	…	…	…	…	…	…	…	…	…
